# Mathematical modelling of activation-induced heterogeneity in TNF, IL6, NOS2, and IL1β expression reveals cell state transitions underpinning macrophage responses to LPS

**DOI:** 10.12688/wellcomeopenres.17557.2

**Published:** 2022-07-25

**Authors:** Shoumit Dey, Dave Boucher, Jon Pitchford, Dimitris Lagos

**Affiliations:** 1 Hull York Medical School, University of York, York, UK; 2 Department of Biology, University of York, York, UK; 3 Department of Mathematics, University of York, York, UK; 4 York Biomedical Research Institute, University of York, York, UK

**Keywords:** mathematical modelling, macrophage heterogeneity, activation induced heterogeneity, TNF, IL6, NOS2, IL1β

## Abstract

**Background: **Despite extensive work on macrophage heterogeneity, the mechanisms driving activation induced heterogeneity (AIH) in macrophages remain poorly understood. Here, we aimed to develop mathematical models to explore theoretical cellular states underpinning the empirically observed responses of macrophages following lipopolysaccharide (LPS) challenge.

**Methods: **We obtained empirical data following primary and secondary responses to LPS in two *in vitro* cellular models (bone marrow-derived macrophages or BMDMs, and RAW 264.7 cells) and single-cell protein measurements for four key inflammatory mediators: TNF, IL-6, pro-IL-1β, and NOS2, and used mathematical modelling to understand heterogeneity.

**Results: **For these four factors, we showed that macrophage community AIH is dependent on LPS dose and that altered AIH kinetics in macrophages responding to a second LPS challenge underpin hypo-responsiveness to LPS. These empirical data can be explained by a mathematical three-state model including negative, positive, and non-responsive states (NRS), but they are also compatible with a four-state model that includes distinct reversibly NRS and non-responsive permanently states (NRPS). Our mathematical model, termed NoRM (Non-Responsive Macrophage) model identifies similarities and differences between BMDM and RAW 264.7 cell responses. In both cell types, transition rates between states in the NoRM model are distinct for each of the tested proteins and, crucially, macrophage hypo-responsiveness is underpinned by changes in transition rates to and from NRS.

**Conclusions: **Overall, we provide a mathematical model for studying macrophage ecology and community dynamics that can be used to elucidate the role of phenotypically negative macrophage populations in AIH and, primary and secondary responses to LPS.

## Introduction

Variability in gene expression in eukaryotic cells is required to allow communities of cells to switch from homeostatic to inducible states while responding to external cues ( Blake *et al.*, 2006; Eldar & Elowitz, 2010). Genetically identical populations show considerable cell-to-cell variability, particularly of proteins that are stress induced ( Bar-Even *et al.*, 2006; Newman *et al.*, 2006). Studies on heterogeneity have found that expression of housekeeping genes tends to be normally (or log normally) distributed in apparently homogeneous populations ( Klein *et al.*, 2015; Kumar *et al.*, 2014) while a subset of genes displays increased cell-to-cell variability with a bi-modal distribution ( Shalek *et al.*, 2013). Population heterogeneity plays a critical role in shaping immune responses. For example, seemingly clonal populations of myeloid cells can produce effector cytokines heterogeneously. Several models of myeloid heterogeneity have been described, including bi-phasic transcription factor activation such as that of NF-kB and autocrine/paracrine effects of TNF or IL-1β in response to TLR stimulation ( Burns *et al.*, 1998; Caldwell *et al.*, 2014; Han *et al.*, 2002; Hayden & Ghosh, 2014) and recently shown to be partly dependent on intercellular desynchronization of molecular clock ( Allen *et al.*, 2019). Interestingly, macrophage hypo-responsiveness to secondary stimulation has been associated with a switch in phenotype wherein, by a combination of TLR4 attenuation, microRNA (miRNA)-mediated silencing expression, and chromatin modifications, macrophages lose their ability to make inflammatory proteins ( Biswas & Lopez-Collazo, 2009; Netea *et al.*, 2015; Seeley & Ghosh, 2017) with alterations in chromatin accessibility being a more permanent cause for this phenomenon. In addition, macrophage hypo-responsiveness is driven by effects only on some genes while expression of others remains unaffected ( Foster *et al.*, 2007). Despite the above insight, the effect of primary or repeated stimulation on macrophage population heterogeneity, termed here as activation-induced heterogeneity (AIH) and the underpinning molecular mechanisms remain elusive.

Here, we aimed to develop a mathematical model capturing AIH within macrophage communities to propose and explore theoretical cellular states underpinning the empirically observed consistency of these communities. We used empirical data from two simple cellular systems of primary and secondary LPS challenge and measuring expression of four pro-inflammatory proteins. Our mathematical models of TNF, IL-6, pro-IL-1β, and NOS2 states reveal that transitions to and from phenotypically negative or non-responding macrophage populations are critical determinants of macrophage AIH and responses to primary and secondary LPS challenge.

## Methods

### Ethics statement

Mouse breeding was performed under a UK Home Office License (project license number PPL 60/4377). Experiments were conducted with approval from the University of York Animal Welfare and Ethical Review Body. Mice were euthanised by CO _2 _inhalation. Animal handling was conducted with care to reduce animal suffering.

### Animals

Female C57BL/6 CD45.2 mice were obtained from Charles River (UK). Animal care was regulated under the Animals (Scientific Procedures) Act 1986 (revised under European Directive 2010/63/EU). Animals were used as a source of tissue only and no procedures were conducted on the animals themselves.

### Study design

In this study, six-eight-week-old female wildtype C57BL/6 CD45.2 mice were used as a source of bone marrow-derived cells. No animal groupings were created prior to bone marrow cell isolation. All animals used in the study were healthy, showing no signs of distress and none were excluded from the study. Animals were kept under specific pathogen-free conditions. Isolated cells from a single animal were differentiated and divided into groups for each independent experimental replicate. The number of animals used in the study was determined based on pilot experiments, and *a priori* sample size calculation was not performed. Results from the study are based on at least three independent experiments and the individual repeat is indicated in figure legends as ‘n’.

### Cell culture

Bone marrow-derived macrophages (BMDMs) were isolated from female C57BL/6 mice and differentiated in the presence of MCSF1 (50ng/ml) for six days and then frozen at -70°C. Frozen BMDM from half a mouse (one tibia and one femur) were plated and cultured in 10mL of macrophage media in 100cm petri dishes for 2–3 days in the presence of MCSF1 before plating them on 24 well plates for experiments.

Both RAW264.7 cells and BMDMs were cultured in Dulbecco’s Modified Eagle Medium (DMEM) supplemented with 1% streptomycin-penicillin mixture, 1% L-glutamine and 10% fetal calf serum (Hyclone). For experiments using BMDM, MCSF1 was added in the cell culture media and kept for the duration of the experiment.

RAW 264.7 cells, are a monocyte/macrophage-like cell line, originating from Abelson leukemia virus transformed cell line derived from BALB/c mice, were detached for passaging using 1x Trypsin-EDTA (Invitrogen) by incubating at 37°C for 10 minutes. Cells were detached completely by gently scraping with cell scraper with a cross-ribbed handle (VWR). Upon reaching 70–80% confluency, cells were harvested and plated in 24 well plates. BMDMs were detached by gentle pipetting up and down using ice cold 1X PBS (Gibco). Cells were centrifuged (1500RPM for RAW264.7 and 1300RPM for BMDMs) at room temperature for five minutes for the purposes of washing or re-suspending.

### LPS challenge

LPS from *Escherichia coli* serotype 055:B5 (Sigma-Aldrich, L2880) was used. This is a phenol extracted LPS with <3% protein impurity. Approximately 125-150,000 RAW264.7 cells or BMDMs were plated overnight before experiments. All cells were plated in a Corning 24 well plate in 500ul of DMEM. For LPS titration experiments, cells were either stimulated with LPS or were left in media (untreated) on day 1. Cells were challenged with 1, 10, 100 or 1000 ng/ml of LPS. Supernatant was collected at 24 hours and stored at -20 ^o^C. Cells were harvested for flow cytometry at 16 or 24 hours from LPS stimulus.

For inducing hypo-responsiveness, cells were either stimulated with 10 or 1000 ng/ml of LPS or left untreated in media on day 0. After 24 hours (day 1), cells were washed twice with PBS and replaced with media (Media/Media) or with media containing 1000 ng/ml of LPS (10/1000; 1000/1000 or Media/1000).

### Flow cytometry

RAW264.7 cells of BMDMs were collected after washing in ice-cold PBS and then detaching the cells with Accutase (BioLegend). Prior to collection, cells were incubated in 10ug/ml of Brefeldin A (BFA, Sigma). BFA was added to the culture four hours prior to harvest for staining.

Cells and all reagents were maintained at 4°C throughout the intra-cellular staining protocol. Harvested cells were washed twice in PBS and re-suspended in approximately 50ul of PBS. Cells were stained with 100ul of 1:1000 Zombie Aqua live/dead stain (BioLegend) in PBS on ice for 8–10 minutes in the dark. F _c_ receptors were blocked with 5ul of 2mg/ml rat IgG for five minutes. Cells were fixed with BD Cytofix and permeabilized with BD Cytoperm. Intracellular staining was performed with the cocktail of antibodies made in permeability buffer. BV421-TNF (MP6-XT22; BioLegend), APC-IL6 (MP5-20F3; BioLegend, eFluor 610-NOS2 (CXNFT; ThermoFisher Scientific), PE-pro-IL-1β (NJTEN3, ThermoFisher Scientific), FITC-F4/80 (BM8, BioLegend) and PE-Cy7 CD11b (M1/70, BioLegend) were used for staining RAW264.7 cells. BMDMs and RAW264.7 cells were pre-gated on live cells, singlets, forward scatter, and side scatter (for gating intact cells), F4/80+ and/or CD11b. Flow cytometry was performed on a BD Fortessa and the analysis done using FlowJo (v10.7.2).

### ELISA and Greiss assays

IL-6, TNF and IL-1β concentrations in the cell culture supernatant were measured by enzyme-linked immunosorbent assay (ELISA) using BioLegend's ELISA MAX Standard. Manufacturer's recommended protocol was followed. Absorbance was read at 450nm with a wavelength correction at 570nm using a VersaMax Microplate Reader (Molecular Devices). Standard curves were generated using four-parameter non-linear fitting to known standard concentrations using SoftMax Pro software (v5.4). Optical density of the unknowns that fit within the linear range of the standard curve was used to calculate the concentration of the sample.

Greiss assay was used to measure nitrite concentrations in the supernatants. Diazotization reaction in Greiss assay was carried out as per manufacturer's instructions (Promega). Plates were read on VersaMax microplate reader capturing absorbance between 520 and 550nm.

### Mathematical modelling

Bespoke MATLAB code, NoRM, was written to implement stochastic simulations using the Doob-Gillespie algorithm. The key transition rates ( *α*, *β*, *β _2_*, *γ*, *γ _2_*) were estimated using rejection sampling according to the following algorithm: the NoRM model was run with 10 ^6^ independently generated parameter sets. Within each parameter set, each parameter ( *α*, *β*, *β _2_*, *γ*, *γ _2_*) was independently sampled from a uniform (U[0.01,1]) distribution. The unitless coefficient *µ* (co-efficient for modelling LPS dynamics) was fixed at 10. The LPS decay rate, *δ*, was fixed at 0.5 *d* ^-1^ to represent an LPS decay rate of approximately 1 day at the highest *in silico* dose (1000ng/ml). To check that the chosen distribution did not influence the results, the process of random parameterisation was repeated with 10 ^6^ independent samples of ( *α*, *β*, *β _2_*, *γ*, *γ _2_*) from normal (N[0.03, 0.01)] and gamma (G[0.68,0.2]) distributions; similarly, alternative choices for the LPS coefficient *µ* were explored, but no qualitative change in model endpoints was observed. Selection of parameter sets that best explained empirical datasets was performed by rejection sampling based on the Akaike Information Criterion (AIC). Finally, to check the sensitivity of the model outcomes (i.e. number of positive, negative, non-responsive or non-responsive permanent cells) to parameterisation, an extended Fourier Amplitude Sampling Test ( Saltelli & Bollardo, 1998) was used to check output variance between input parameters using the R based SPARTAN tool ( Alden *et al*., 2013): briefly, parameter sets were sampled from the parameter space using sinusoidal functions in a way that the frequency of the parameter of interest is varied more than the others. A total of 3 resample curves were used to select 65 parameter sets chosen along the curve for each of the 7 parameters (NoRM model) plus one dummy parameter resulting in a total of 1560 parameter sets. Each parameter set was then used to run the model a further 30 times to account for the noise associated with the Doob-Gillespie implementation.

***Modelling process.*** The following section describes the modelling process in detail. In particular, it describes simpler models that were tested to arrive at our proposed model for macrophage activation.

***Positive-state model definition.*** The response to LPS in the *in silico* cell environment was modelled as a direct positive effect on the forward rate that describes the transition of a cell to the P state such that


$$\alpha_{LPS}=\alpha (1+\mu L)\;\text{–}\;equation\;S1$$

where *α _LPS_* is the overall forward rate which takes into account the LPS in the environment, *α* is the forward rate in the absence of LPS, *L* is the concentration of LPS in the environment and *µ* is a constant that describes the magnitude of the response to LPS concentration. The linear assumption in *equation S*1 is used for simplicity to induce plausible local LPS dynamics at the cost of introducing a single unknown, *µ*.

Upon describing the above model as an ODE, we have


$$\frac{dP}{dt}=N\alpha (1+\mu L)-\beta P\;\text{–}\;equation\;S2$$

where $\frac{dP}{dt}$ represents rate of change in the number of cells in the positive state with respect to time, *P*, *N* are the number of *in silico* cells in the positive and negative state respectively, *β* is the rate at which cells in the positive state change to negative state.

We model the decay of LPS concentration, *L* as a simple first-order exponential decay in continuous time. This can then be expressed as


$$L(t)=L_{0}e^{-\delta t}\;\text{–}\;equation\;S3$$

where *L*_0_ and *L*( *t*) represent the concentration of LPS at time zero and *t* respectively, and *δ* is the constant LPS decay rate.

At a time, dependent quasi-equilibrium, where the dynamics of *L* are assumed to occur on a slower time scale than those of *P* and *N*, equation S2 can be used to write


$$P_{quasi}^{*}=\frac{N\alpha (1+L_{0}e^{-\delta t})}{\beta }\;\text{–}\;equation\;S4$$

where rate of change of *P*, $\frac{dP}{dt}$ = 0 and $P_{quasi}^{*}$ represents the number of *in silico* cells in the positive state at quasi-equilibrium.

It can be inferred from equation S4 that in the absence of LPS in the environment,


$$P^{*}=\frac{N\alpha }{\beta }\;\text{–}\;equation\;S5$$

which is the equilibrium ( P*) dynamics for the simple case where two species switch between each other with rates *α* and *β* respectively.

Further, equation S2 can be re-written as


$$\frac{dP}{dt}=(T-P)\alpha (1+\mu L)-\beta P\;\text{–}\;equation\;S6$$

where *T* is total number of cells since *T* = *N * + *P* at any given time.

Equation S6 can be solved exactly to the following closed form equation given the initial condition *P*_(0)_ = 0.


$$P_{\left(t\right)}=e^{\frac{L_{0}\alpha \mu e^{-\delta t}}{\delta }-t(\alpha +\beta )}\int_{0}^{t}T\alpha e^{\alpha t+\beta t-(L_{0}\alpha \mu e^{-\delta t})/\delta }(L_{0}\mu e^{-\delta t+1})dt\;– equation S7$$

While equation S7 can model the fraction of macrophages responding to a primary LPS stimulation, it fails to explain the effect of second dose of LPS wherein a smaller fraction of the population responds unless the value of *α* is modified for the population. This implies that the LPS response of the whole population changes upon secondary stimulus and is incompatible with experimental results, at the transcript level, cytokines are expressed bi-modally ( Shalek *et al*., 2013).

***Non-responsive state model (“three-state model”).*** We then explored whether the inclusion of an additional state (phenotype) could explain macrophage hypo-responsiveness with respect to any one protein. To this effect, we first excluded the LPS-induced effect on *α* and explored simpler linear explanations of what we observe by considering the following equations


$$\frac{dN}{dt}=-N\alpha_{LPS}+\beta P+\beta_{2}NR\;\text{–}\;equation\;S8$$

$$\frac{dP}{dt}=N\alpha_{LPS}-\beta P-\gamma_{1}P\;– equation S9$$

$$\frac{dNR}{dt}=\gamma_{1}P-\beta_{2}NR\;\text{–}\;equation\;S10$$

where *NR* represents the non-responsive state and _1_ represents the rate at which positive cells change to a non-responsive state ( *NR*, equation S10) while *β*_2_ rate at which non-responsive cells become negative cells ( *N*, equation.S8) and *α _LPS_* is LPS dependent ( equation S1).

***Non-responsive state model (“four-state model”).*** We then asked whether the inclusion of a fourth state (a non-responsive permanent state or NRPS) can resolve empirical hypo-responsiveness better. NRPS state is modelled as an end-point state in the model with the rate of change equation for NRPS state and modifying equation S10 as follow:


$$\frac{dNRPS}{dt}=\gamma_{2}NR\;\text{–}\;equation\;S11$$

$$\frac{dNR}{dt}=\gamma_{1}P-\beta_{2}NR-\gamma_{2}NR\;\text{–}\;equation\;S10$$

where _2_ is the rate at which cells become permanently non-responsive.

### Statistics

All experiments were performed in at least three biological replicates. BMDM experiments were performed with macrophages from at least three mice. Statistical and numerical analysis was done using Graphpad Prism (version 9.3.1) and Matlab (R2019a). Treatment groups were compared by multiple comparisons one-way ANOVA. The level of significance was set at 0.05.

## Results

### Macrophage community AIH is dependent on LPS dose

To obtain empirical data for our mathematical model and to capture distinct macrophage subpopulations upon activation with LPS we measured protein expression of three cytokines, TNF, pro-IL-1β and IL-6, and one intracellular pro-inflammatory protein NOS2, an enzyme that catalyzes nitric oxide formation. We selected these factors as they are all inducible upon LPS challenge. Furthermore, heterogeneity in TNF and IL-1β secretion in populations can be a result of bi-phasic NF-kB activation ( Tay *et al.*, 2010). IL-6 and NOS2 are also up-regulated due to LPS ( Farlik *et al.*, 2010; Tanabe *et al.*, 2010). In addition, all these proteins have been implicated in LPS-induced macrophage hypo-responsiveness. To study AIH, we focused on the early stages (within the first 24h) post-primary or secondary stimulation with LPS to minimise confounding effects of secondary and tertiary cytokine-mediated effects.

**Figure 1.**
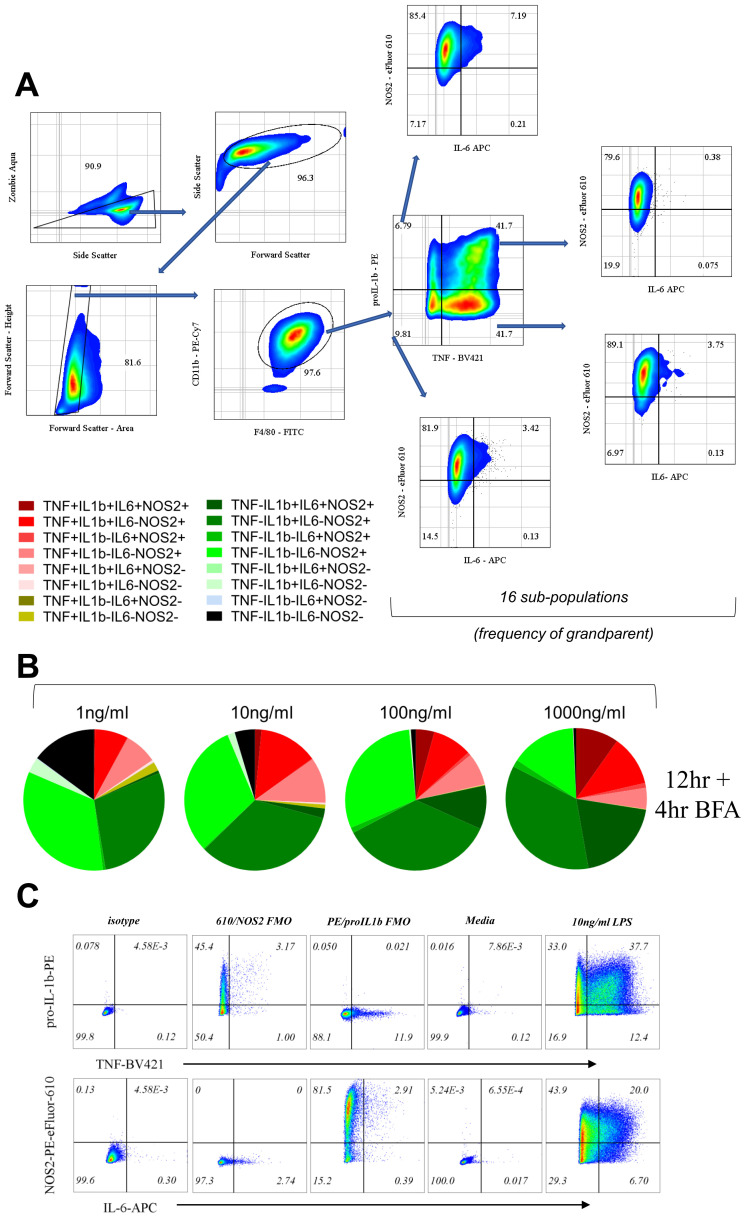
Macrophage community AIH is dependent on LPS dose. **A**. Flow cytometry gating to show 16 sub-populations determined based on TNF, IL-6, pro-IL-1b and NOS2. **B**. Pie charts represent the community composition at 16 hours post stimulus with the indicated doses of LPS for RAW264.7. Data representative of three independent experiments. **C**. Bi-plots describing strategy for gating for positive and negative fractions for TNF, pro-IL1β, IL-6 and NOS2 positive cells throughout the experiments shown here for BMDMs as a representation. Gating was adjusted based on pooled isotype and re-confirmed using fluorescent minus one controls (FMOs for PE and PE-eFluor 610) with controls for media only treatment. Finally, a representative graph showing cells treated with 10ng/ml LPS to show staining example and positive fractions.

First, we selected RAW264.7 cells as a cellular model. These cells are thought to be a model of primary bone-marrow derived macrophages with regards to expression of surface receptors and the response to microbial ligands ( Berghaus *et al.*, 2010). We reasoned that using a macrophage cell line to study AIH also reduced the level of starting population heterogeneity in comparison to that we would observe using primary macrophages. The community composition of LPS-stimulated RAW264.7 cells was represented graphically by charting the 16 possible sub-populations by adapting the Simplified Presentation of Incredibly Complex Evaluations (SPICE) method ( Roederer *et al.*, 2011), with each slice representing a subpopulation ( Figure 1A, B) with positive fractions selected based on appropriate isotype controls (supplementary Figure 1C). Consistent with the concept of AIH, we found that the dose of LPS can have qualitative effects on the diversity of the response; quadruple positive and TNF negative triple positive (TNF-proIL1β+IL6+NOS2+) cells appear prominently at higher doses of LPS (100, 1000ng/ml; Figure 1B), while quadruple negative (TNF-pro-IL1β-IL6-NOS2-) sub-populations and single positive cells for TNF (TNF+pro-IL1β-IL6-NOS2-) appear at lower doses (1, 10ng/ml; Figure 1B). Despite heterogeneous compositions of low and high dose of LPS, double-positive TNF-proIL1β+IL6-NOS2+ cells were a part of all LPS doses with little variability (1, 10, 100, 1000ng/ml; Figure 1B) suggesting the presence of sub-populations with differential dependence on the magnitude of LPS dose.

**Figure 2.**
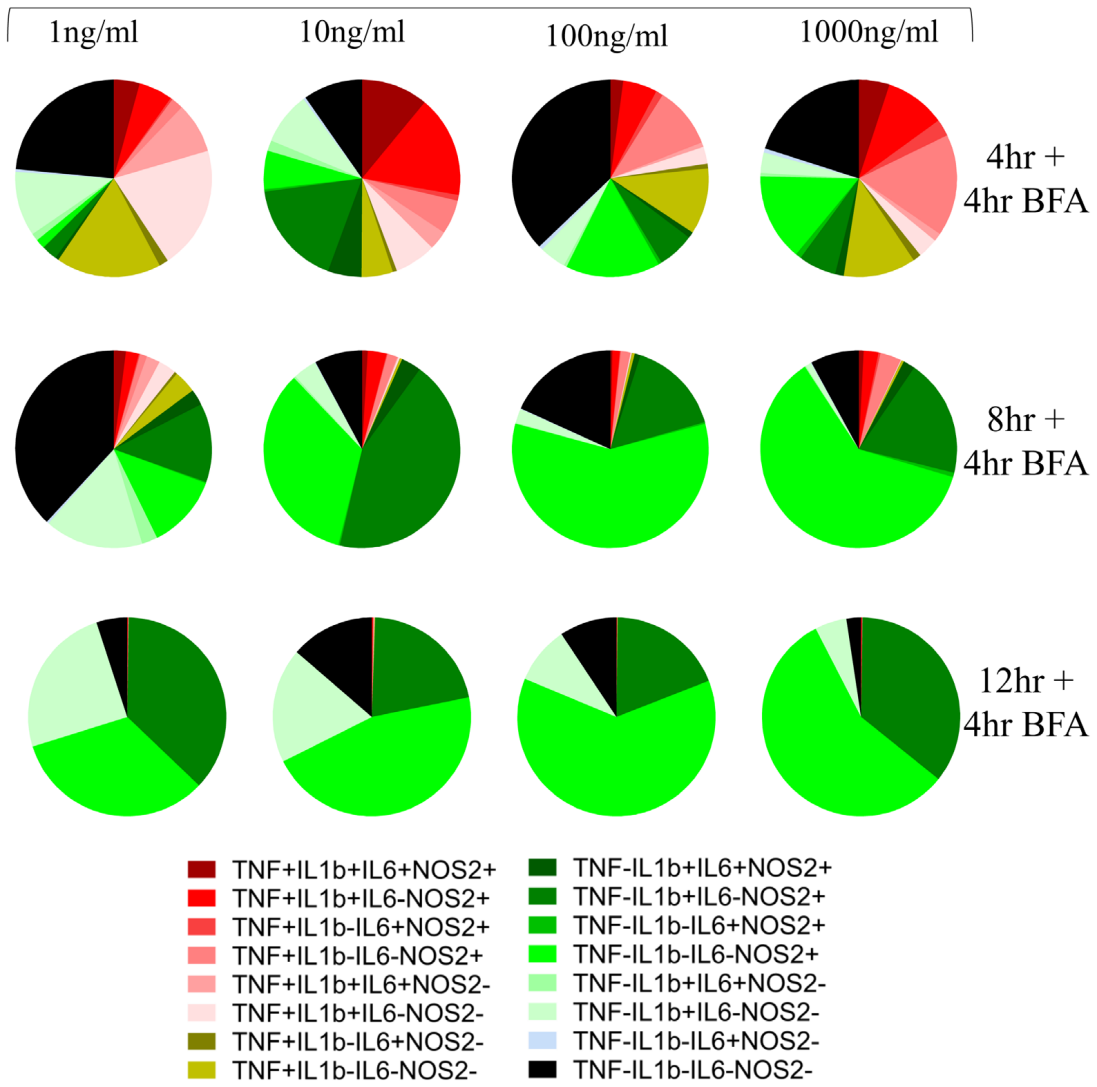
Macrophage community AIH kinetics for BMDMs. Pie charts represent the community composition at 8, 12 and 16 hours post stimulus with the indicated doses of LPS for BMDMs. BMDMs are pre-gated on Live/Singlets/FSC-SSC/CD11b+F4/80+ population. Data representative of three independent experiments.

Next, we explored AIH in BMDMs, a cell model more faithfully capturing heterogeneity of primary macrophages. As in the case of RAW264.7 cells, exposure to LPS induced population heterogeneity in BMDMs albeit with different kinetics to that observed in RAW264.7 cells (compare Figure 2 with Figure 1B). Whereas all populations were observed at 16h (12h stimulation followed by 4h of BFA treatment) post-stimulation in RAW264.7 cells, in BMDMs this was the case at earlier timepoints but not at 16h. Notably, the percentage of TNF-positive BMDMs peaked at 4h post stimulation, demonstrating a faster TNF response in BMDMs in comparison to RAW264.7 cells. In BMDMs, single positive cells for NOS2+ cells (TNF-pro-IL1β-IL6-NOS2+) increased while single positive cells for pro-IL1β (TNF-pro-IL1β+IL6-NOS2-) decreased with increasing magnitude of LPS dose at all time points ( Figure 2). Further, quadruple negative sub-population (TNF-pro-IL1β-IL6-NOS2-) did not show a clear increase with a lower LPS dose as in RAW264.7 cells suggesting that the appearance of these sub-populations is more nuanced in BMDMs. While higher frequency of quadruple negative cells in 1ng/ml versus 10ng/ml could reflect differences in responses to increasing amounts of LPS the increased quadruple negative sub-population frequency in 100 and 1000ng/ml concentration may be due to a fast response accompanied by an immediate switch to a non-responding phenotype.

Overall, our findings indicated that exposure to LPS induced population heterogeneity in macrophage communities for both a macrophage cell line (RAW264.7 cells) and primary BMDMs. As expected, cell-type specific differences were observed with BMDM responses occurring and peaking faster and reaching a plateau at lower LPS concentrations. These could be linked to differential sensitivity to LPS, but also differential pre-existing population heterogeneity between BMDMs and RAW264.7 cells. Regardless of these differences in kinetics our findings demonstrated that upon primary LPS challenge, AIH occurs in macrophages in an LPS-dependent manner. 

### Altered AIH kinetics in response to a second LPS challenge underpin macrophage hypo-responsiveness

**Figure 3.**
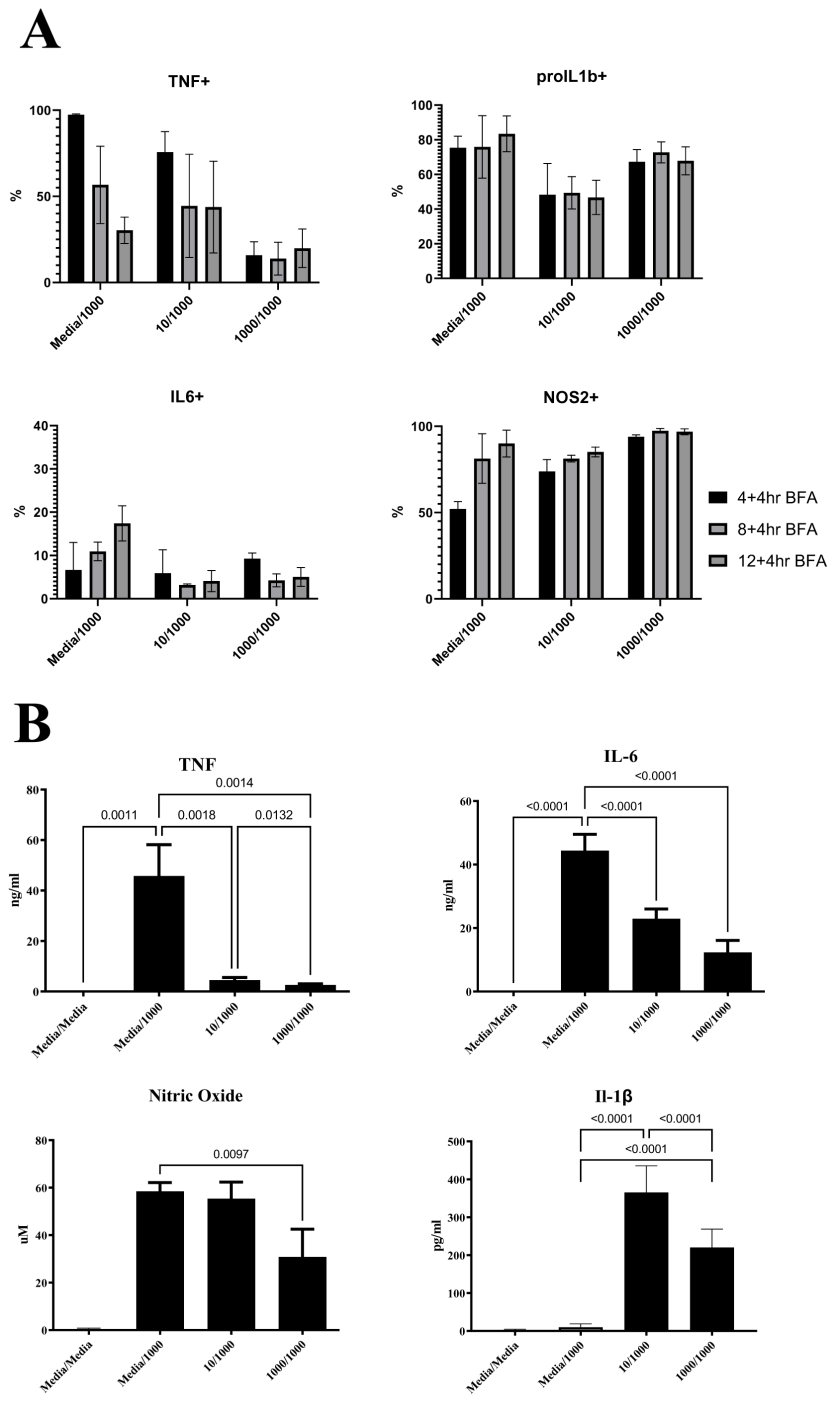
Altered cytokine production kinetics in RAW264.7 macrophages responding to a second LPS challenge. Bar plots indicating mean and error bars show standard deviation of **A**) Percentages for TNF, pro-IL1β, IL-6 and NOS2 positive cells at the indicated timepoints post LPS challenge (1000ng/ml) in macrophages pre-treated for 24 hours with either media (Media/1000), or 10ng/ml LPS (10/1000), or 1000ng/ml LPS (1000/1000) for RAW264.7 cells. **B**) TNF, IL-6, IL-1β and Nitric Oxide (NO) cumulative secreted levels at 24 hours post-secondary stimulation with 1000ng/ml LPS in macrophages pre-treated for 24 hours with either media (Media/1000), or 10ng/ml LPS (10/1000), or 1000ng/ml LPS (1000/1000). n=6 per treatment group.

**Figure 4.**
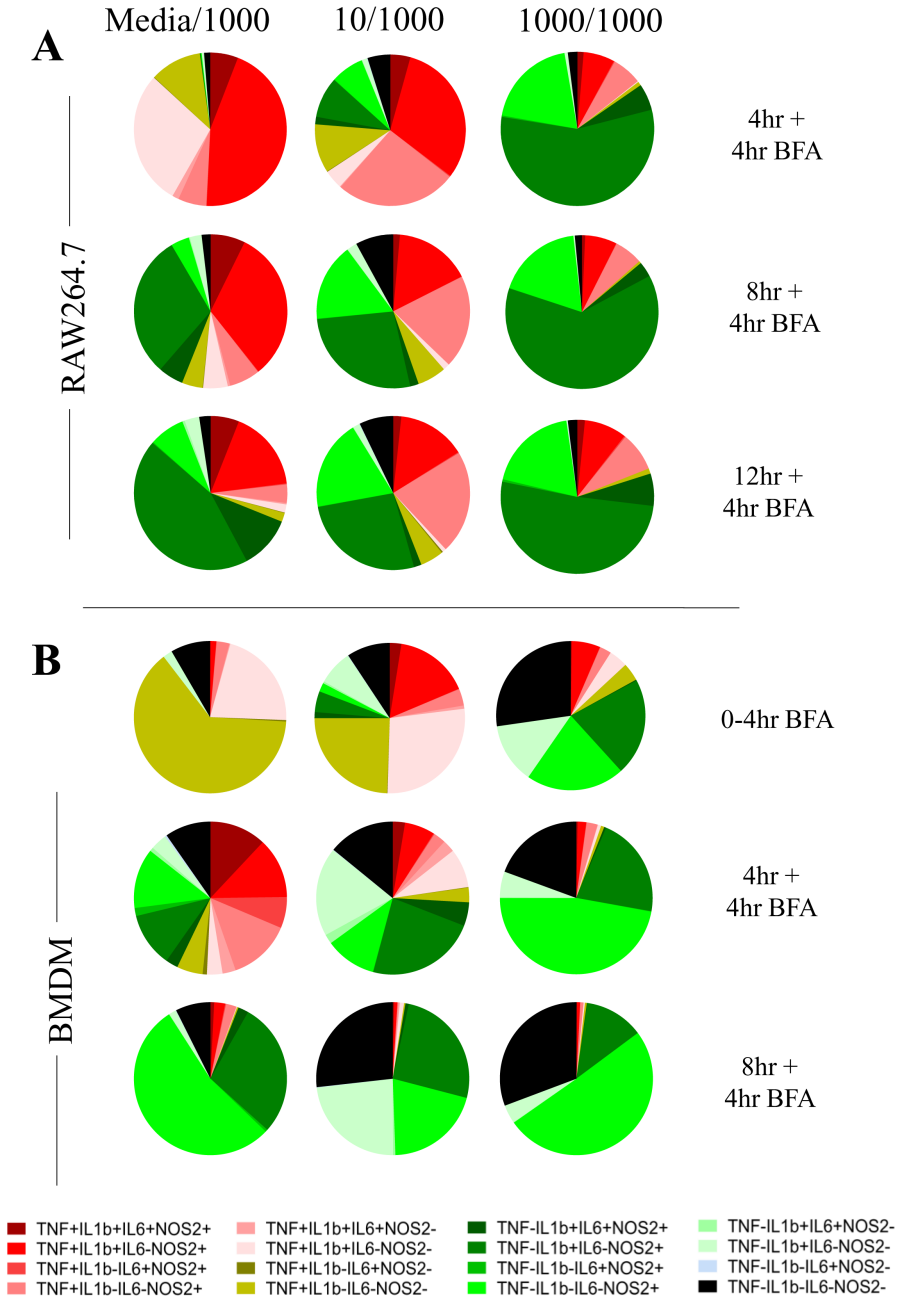
Altered AIH kinetics in macrophages responding to a second LPS challenge correlate with hypo-responsiveness. **A**. Averaged pie charts representing n=3 independent experiments at the indicated timepoints post LPS challenge (1000ng/ml) of RAW264.7 macrophages pre-treated for 24 hours with either media (Media/1000), or 10ng/ml LPS (10/1000), or 1000ng/ml LPS (1000/1000). Legend indicates expression status for TNF, IL-6, pro-IL-1b and NOS2 subset. **B**. Same as above but for BMDMs. n=3 for 0-4hr BFA, n=4 for 4+4hr BFA and n=2 for 8+4hr BFA

Next, we tested how changes in macrophage community compositions in RAW264.7 cells compared between macrophages challenged with LPS for a second time and macrophages responding to a first LPS stimulus. We obtained temporal snapshots of RAW264.7 cell communities responding to LPS alongside LPS responses of communities that were pre-exposed to varying LPS doses ( Figure 3A). At the population level, cumulative secreted levels of TNF, IL-6, and NO were reduced for RAW264.7 cells ( Figure 3B), supporting that the LPS pre-treatment compromised the ability of cells to respond to a second LPS challenge. At the community level, single-cell measurements revealed that pre-treated macrophage community consistency (10/1000 and 1000/1000) differed to that seen during primary challenge (Media/1000) at 8h and 12h post stimulation but not at 16h ( Figure 4). For example, pre-treated macrophages were characterised by a prominent TNF-pro-IL1β+IL6-NOS2+ population but reduced TNF+ populations at the earlier stages of the response. This suggested that LPS-induced hypo-responsiveness is underpinned by different starting community compositions and altered community evolution trajectories, but not a different endpoint community composition.

**Figure 5.**
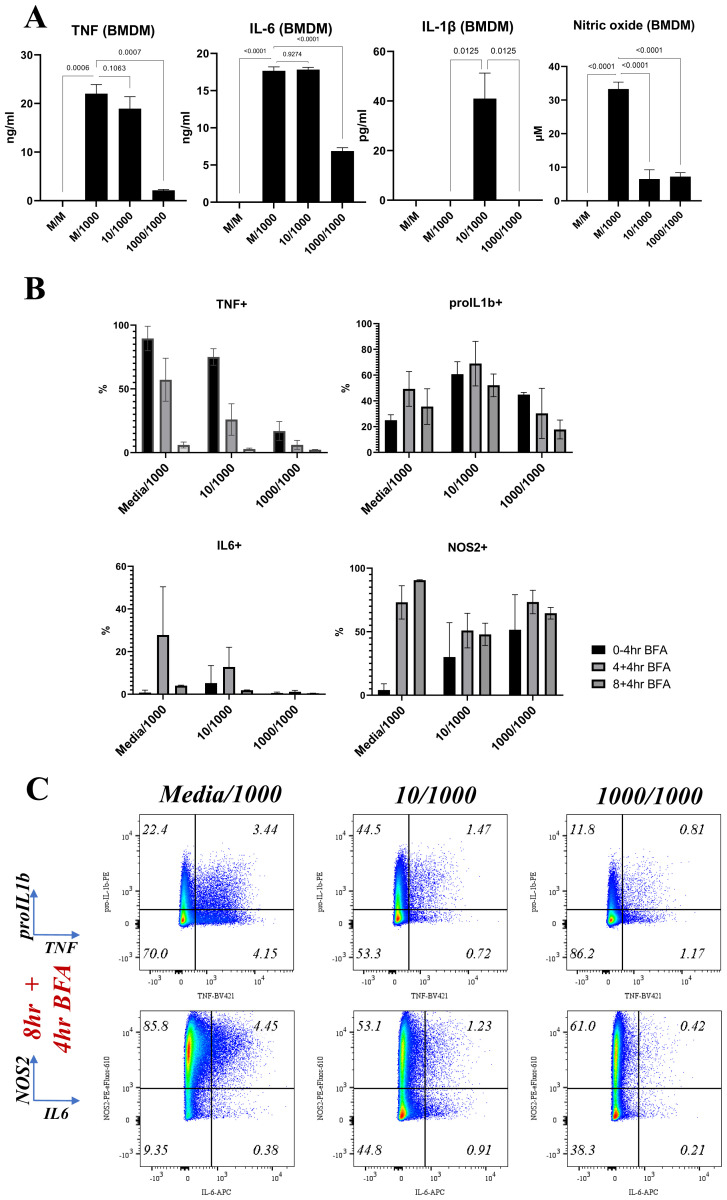
BMDMs show a clear hyporesponsive phenotype by flow. Bar plots indicating mean and error bars show standard deviation of **A**. TNF, IL-6, IL-1β and Nitric Oxide (NO) cumulative secreted levels at 24 hours post-secondary stimulation with 1000ng/ml LPS in macrophages pre-treated for 24 hours with either media (Media/1000), or 10ng/ml LPS (10/1000), or 1000ng/ml LPS (1000/1000). n=4 per treatment group **B**. Percentages for TNF, pro-IL1β, IL-6 and NOS2 positive cells at the indicated timepoints post LPS challenge (1000ng/ml) in BMDMs pre-treated for 24 hours with either media (Media/1000), or 10ng/ml LPS (10/1000), or 1000ng/ml LPS (1000/1000) and **C**. Bi-plots showing TNF, pro-IL-1β and IL-6, NOS2 expression at indicated timepoints post LPS challenge (1000ng/ml) in BMDMs pre-treated for 24 hours with either media (Media/1000), or 10ng/ml LPS (10/1000), or 1000ng/ml LPS (1000/1000). Cells were pre-gated on Live/Singlets/FSC-SSC-A/CD11b+F4/80+. Plots representative of 3 independent experiments

In BMDMs, LPS-induced hypo-responsiveness was observed for cells pre-treated with 1000ng LPS for cumulative secreted levels of TNF, IL6, NO, and IL1β ( Figure 5A). IL1β secretion was only observed in BMDMs pre-treated with 10ng LPS, in agreement with the known requirement of a priming step for pro-IL1β processing and IL1β secretion ( Eder, 2009; Lopez-Castejon & Brough, 2011). BMDMs pre-treated with 1000ng LPS failed to produce secreted IL1β ( Figure 5A), further supporting their hypo-responsive phenotype. At the single-cell level, despite increased levels at early timepoints (0–4hr) for NOS2 and pro-IL1β, we observed reduced expression of all measured proteins at 12 hours post stimulation of BMDMs pre-treated with 1000ng LPS ( Figure 5B, C) and increased quadruple negative population at all timepoints ( Figure 4B). Having observed the kinetic differences between RAW264.7 cells and BMDMs upon primary LPS challenge ( Figure 1B and Figure 2), we explored an earlier time point in BMDM response (0–4hr in BFA, Figure 4B). Indeed, 90% of all BMDMs also undergo an TNF+ state after which a fraction continues to be in the TNF+ sub-populations and a fraction that switches off (0–4hr BFA versus 4hr +4hr BFA in Media/1000 group). This finding is also in line with TNF being an early response protein ( Bradley, 2008) and shaping macrophage community structure ( Caldwell *et al.*, 2014). As in the case of RAW264.7 cells, we observed more striking community differences during the early timepoints of the response (4hr+4hr BFA and 8h+4hr BFA) between pre-treated and control BMDMs. The end-point compositions (8h+4hr BFA) were less distinct, although we note that in BMDMs, LPS pre-treatment resulted in an increase in quadruple negative cell populations and a reduction in TNF+ populations in LPS-pretreated cells at 12h post challenge ( Figure 4B).

In the 10/1000 community 76% RAW264.7 cells (4hr+4hr BFA, Figure 4A; 75% BMDMs at 0–4hr, Figure 4B) were positive for TNF and hypo-responsiveness was most pronounced in the 1000/1000 community with just 16.9% BMDMs and 18% RAW264.7 cells being TNF+ in the first four and eight hours of the response respectively ( Figure 4). Furthermore, in both BMDMs and RAW264.7 cells and at the earliest timepoints, the 10/1000 community showed a higher percentage of TNF+ cells than the 1000/1000 community that switch off rapidly to 45% for RAW264.7 cells and 26% for BMDMs at 12 and 8 hours respectively, suggesting that a lower dose pre-stimulus decreases the capability of a population of cells to switch on TNF in response to a higher dose pre-stimulus. Furthermore, RAW264.7 communities of 10/1000 and 1000/1000 comprised of 5% and 2% negative sub-population respectively, confirming again that a small percentage of cells do not respond to the second dose of LPS. In addition, while overall TNF+ cells decrease over eight, 12 and 16 hours post LPS stimulus, the numbers of overall TNF+ cells first decrease (between eight and 12 hours) then increase (between 12 and 16 hours) in RAW264.7 1000/1000 communities ( Figure 4A). This suggested that a subset of cells can become positive for TNF later in response to the secondary stimulus.

Interestingly, in BMDMs it was the quadruple negative (TNF-pro-IL1β-IL6-NOS2-), single positive NOS2 sub-population (TNF-pro-IL1β-IL6-NOS2+) and the double positive sub-population TNF-pro-IL1β+IL6-NOS2+ that dominated (27.3%, 21.4% and 21% respectively, Figure 4B 0–4hr) the first 4 hours of response in the 1000/1000 community compared with the Media/1000 community ( Figure 4B 4hr). Similar to BMDMs, in RAW264.7 cells, the 1000/1000 community response in the first 8 hours also comprised of single positive NOS2 (20%, 4+4hr BFA Figure 4A) and double positive TNF-pro-IL1β+IL6-NOS2+ (57%, 4+4hr BFA Figure 4A). IL-6+ sub-populations were consistently reduced at all timepoints when compared between the Media/1000 and the 1000/1000 communities ( Figure 4B, Figure 5B, C).

Overall, these results demonstrated that for both RAW264.7 cells and BMDMs, pre-exposure of macrophages to low or high LPS doses resulted in altered AIH kinetics during a secondary LPS challenge in comparison to macrophages receiving a primary LPS challenge. Endpoint community compositions showed modest differences between cells responding to one or two LPS challenges. The observed secreted protein hypo-responsiveness phenotype was predominantly reflected in the altered kinetics of changes in community composition upon LPS stimulation. Our data indicated that a critical part of the community response to LPS occurred in the first 8–12 hours for RAW264.7 cells and 4–8 hours for BMDMs of the primary challenge, suggesting that at later time points a proportion of cells might be non-responsive in a reversible or permanent manner. We note that the effects were different for each of the measured proteins ( Figure 4), suggesting that protein-specific mechanisms were involved in LPS-induced hypo-responsiveness.

### Transitions between distinct non-responding macrophage subsets underpin responses to LPS

**Figure 6.**
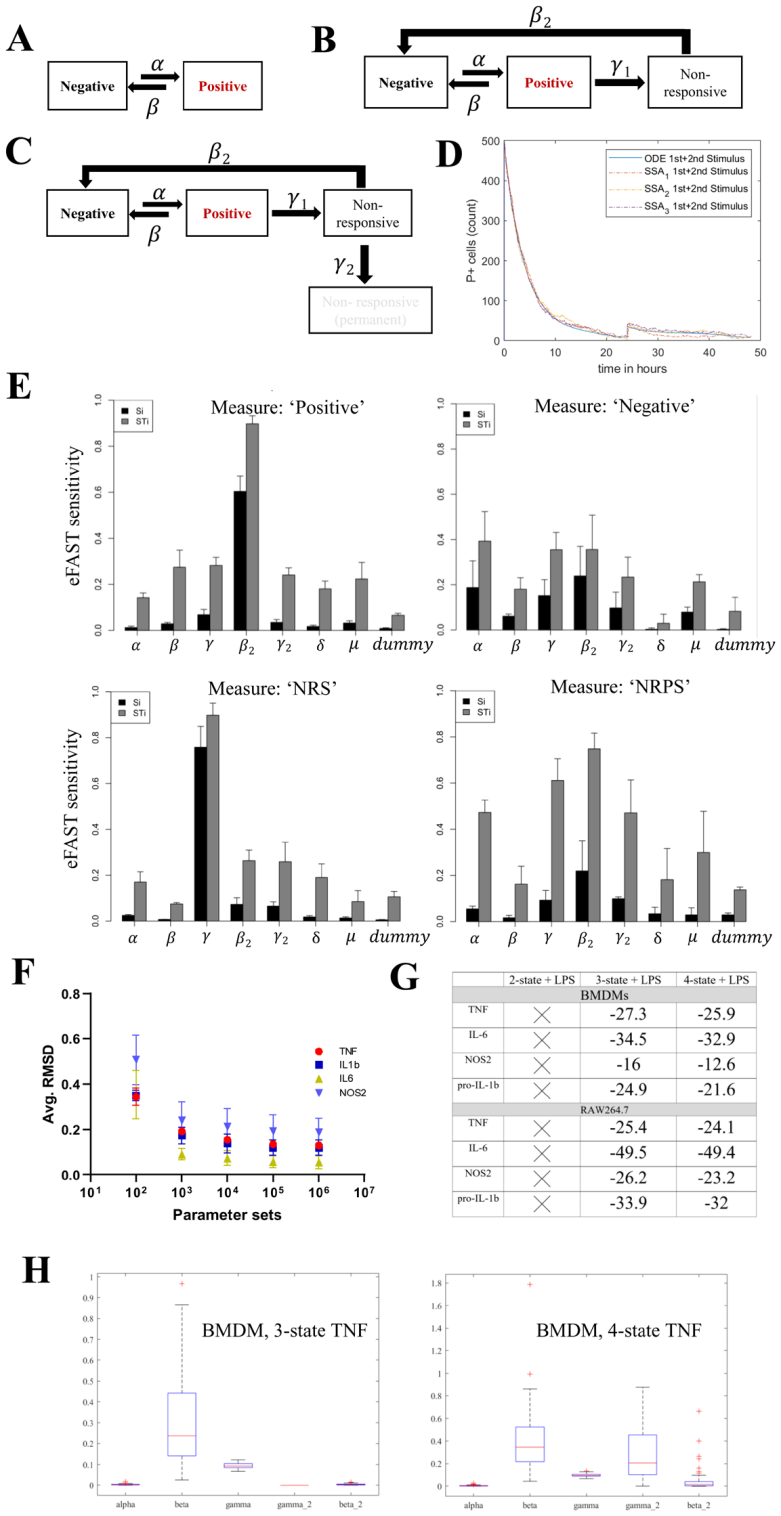
Mathematical modelling with 3 or 4 non-responsive states (NoRM) can describe hypo-responsiveness. **A**. Schematic representation of states and constants used in a 2-state model. Macrophage can be in a negative or positive state of making an inflammatory response protein. **B**. Schematic representation of states and constants used in the NoRM mathematical model. Macrophage can be in a negative, positive, non-responsive (NRS). **C**. Same as B but with inclusion of a 4 ^th^ non-responsive permanent (NRPS) state. **D**. Examples of the bespoke Doob-Gillespie algorithm for NoRM model simulations (n=3) overlaid on the mean-field ODE based non-responsive model with 3-states to show consistency between deterministic and stochastic approaches. Time=0hr represents first *in silico* LPS stimulation and time=24hr represents second LPS stimulation. **E**. Partitioning of Variance in Simulation Results using eFAST: Barplots showing first order (Si) and higher order (STi) sensitivity representing model effects due to the parameter or non-linear effects of the parameter (ie interaction with other parameters are depicted) for 4 different simulation outputs, namely, positive, negative, non-responsive (NRS) and non-responsive permanent (NRPS) states. **F**. Dot plot depicting the reduction of average RMDS for all four proteins as higher number of parameter sets were sampled. **G**. Table depicting average Akaike Information Criterion (AIC) values obtained for top 50 estimated parameter sets 3 versus 4 state NoRM model per protein. AIC was not estimated for 2-state model. **H**. Box and whiskers plots representing the top 50 parameter sets and their corresponding values that fits empirical data for TNF in BMDMs in a 3/4-state model.

The above empirical studies formed the foundation for constructing conceptual mathematical models to understand how AIH contributes towards macrophage responses to LPS. At the heart of these models is the idea that any individual cell may make a transition from a non-protein producing state to a protein producing state (termed “negative” and “positive” states hereafter). These transitions occur at random in continuous time, and the probability (per unit time) of transition depends on the current environment of a cell such as the presence or absence of antigen ( Figure 6A and Methods: Modelling process). The simplest models restricted each cell to be in a negative or a positive state only. While these models were found to be useful to understand antigen (LPS)-dependent switching on and off for each individual protein independently ( Equation 7, Methods: Modelling process), they failed to describe the ability of a subset of cells to become hypo-responsive that was suggested by our empirical studies without explicitly changing the rate at which a negative population switched to positive (Methods: Modelling process). Therefore, we refined the model by allowing two further cell states which reflect the empirical observations. Explicitly, we allowed the possibility that a positive cell could switch to a third non-responsive state (NRS, Figure 6B), generating a three-state model. In addition, we also explored the possibility that cells in the NRS may make one of two transitions, either to a fourth, non-responsive permanently state (NRPS, Figure 6C) or back to the negative cell state, generating a four-state model. Due to variability in experimental data (see Figure 2 versus 4 for M/1000 at 4hr+4hr BFA) we further modelled the above ODE model using the Doob-Gillespie algorithm to account for interpreting variability as stochastic noise (NoRM model). We first checked if the NoRM model and ODE model agree in terms of the latter representing a mean-field for the stochastic model ( Figure 6D). Next, we calculated the eFAST sensitivity each of our model outputs (i.e. number of positive, negative, NRS and NRPS population) by perturbing one parameter at a time ( Saltelli & Bollardo, 1998). We find that variability of the model is affected mostly by the rate parameters ( Figure 6E). We find that the number of ‘positive’ and ‘NRPS’ cells is mostly affected by *β _2_* while ‘NRS’ cells were most affected by *γ* ( Figure 6E). We summarise the best fitting parameter sets identified by our rejection sampling method, and justify the choice of 10 ^6^ independent samples, in Figure 6F; the consistency with our experimental data is displayed in Figure 6G. As an example, the parameter sets which best explain the empirical endpoints for TNF are summarised in Figure 6H.

We termed our overall modelling approach the “non-responsive macrophage” (NoRM) model ( Figure 6). We stress that the purpose of these models was not to predict detailed physiological transitions or identify mechanisms. Rather, they offered a framework within which to interpret our empirical datasets and alluded to simple explanations for observed phenomena across a range of experimental conditions. In this context, we note that in the NoRM model, all cells were expected to respond to LPS treatment. This assumption also captured cells that might never respond to LPS by transitioning from positive to non-responsive states almost immediately upon stimulus.

### A three-state NoRM model is sufficient to explain macrophage hypo-responsiveness

**Figure 7.**
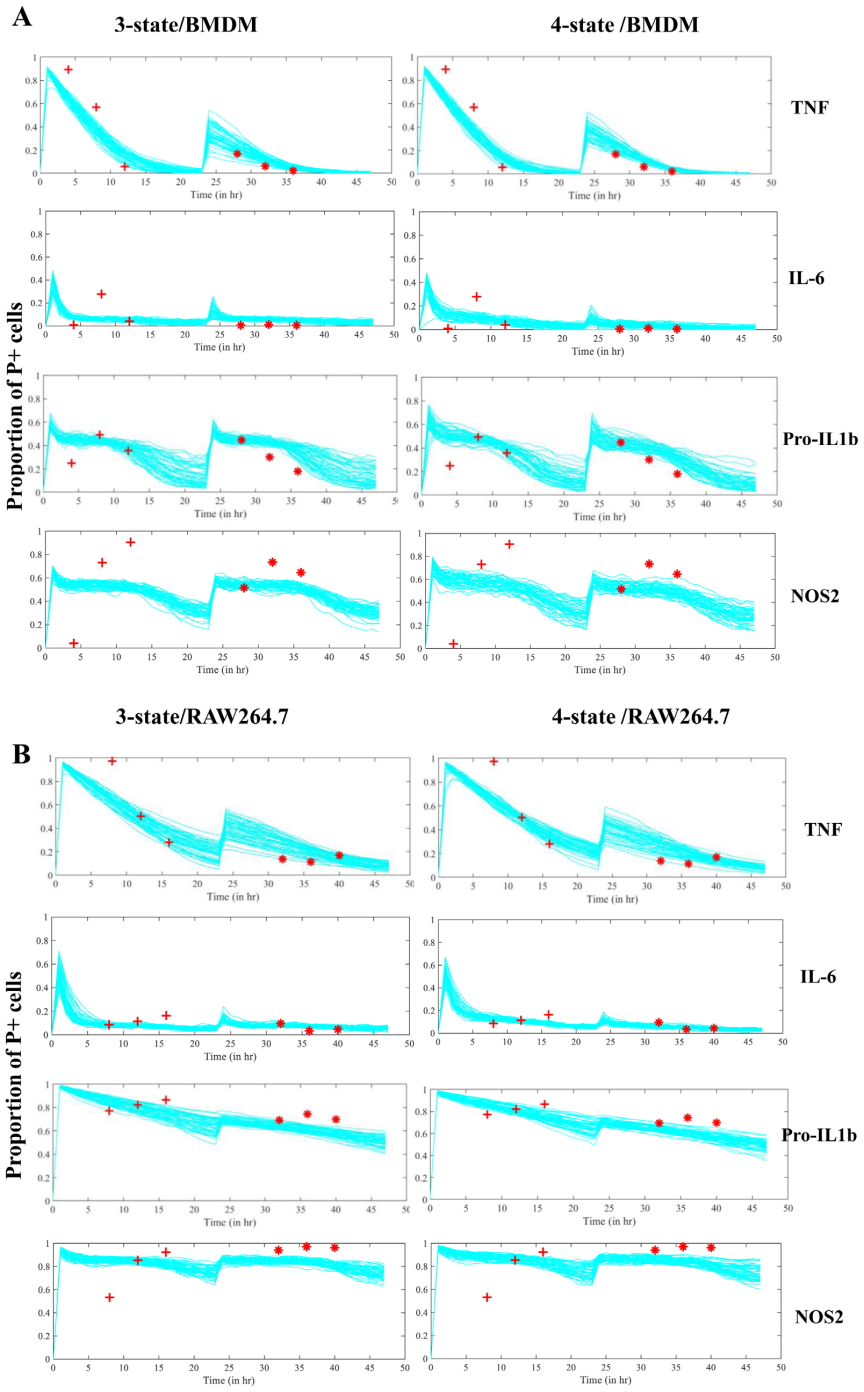
Estimated trajectories of 3 and 4-state NoRM model. **A**. Two LPS stimulations are simulated using the NoRM models3-state left, 4-state right) using top 50 estimated parameter sets (lowest AIC)) based on experimental timepoints marked in red. Time=0hr represents first *in silico* LPS stimulation and time=24hr represents second LPS stimulation for BMDMs **B**. Same as above but for RAW264.7 cells

**Figure 8.**
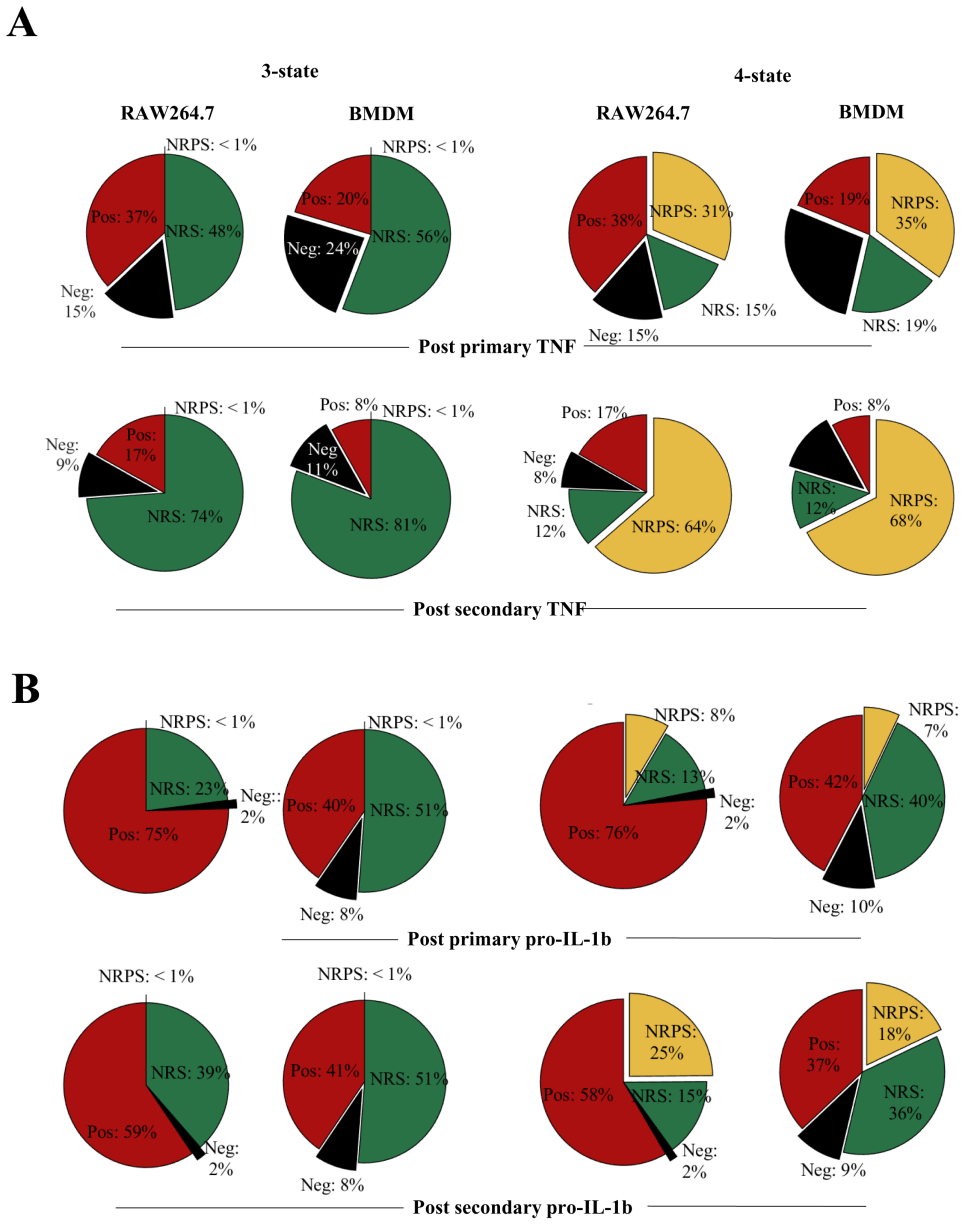
Transitions between distinct non-responding macrophage subsets underpin responses to LPS. **A**. Overall cell-state compositions for TNF based on the NoRM model prediction when 3-states (ie γ _2_=0) or 4-states are modelled post *in silico* stimulation with a single dose of LPS of 1000ng/ml, 12 hours BMDM or 16 hours RAW264.7) and two doses of LPS (1000ng/ml 0-24 hours + 1000ng/ml, 12 hours BMDM or 16 hours RAW264.7). **B**. Same as above but for pro-IL-1b.

**Figure 9.**
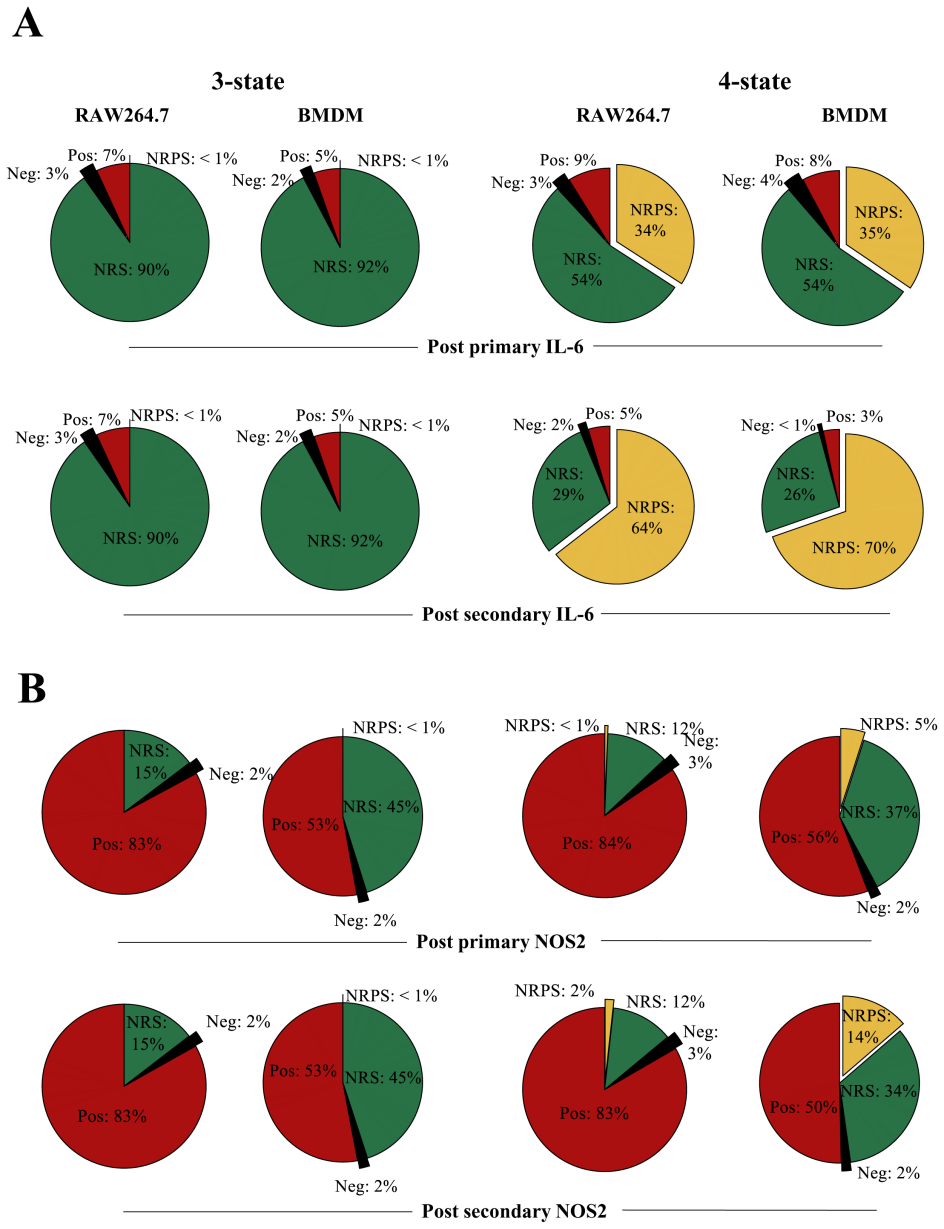
Transitions between distinct non-responding macrophage subsets underpin responses to LPS. **A**. Overall cell-state compositions for TNF based on the NoRM model prediction when 3-states (ie γ _2_=0) or 4-states are modelled post *in silico* stimulation with a single dose of LPS of 1000ng/ml, 12 hours BMDM or 16 hours RAW264.7) and two doses of LPS (1000ng/ml 0-24 hours + 1000ng/ml, 12 hours BMDM or 16 hours RAW264.7). **B**. Same as above but for pro-IL-1b.

Using rejection sampling, we tested whether the three- or four-state NoRM models could independently capture our empirical data for each of the measured proteins. Based on the AIC values comparing model fit to estimated parameters, a three-state NoRM model is sufficient to explain our empirical data ( Figure 6E). We next compared model outputs for proportion of cells in the positive state over time for the three-state and four-state NoRM model both of which predict hypo-responsiveness of the population ( Figure 7). The output from the models was used to predict the composition of positive, negative, NRS and/or, in the case of the four-state model, NRPS for each of the four proteins. Based on the estimated parameters, our model predicted that the total proportion of non-responsive cell-states (NRS and/or NRPS) increased post primary LPS stimulus ( Figure 8, Figure 9) and therefore contributed to the diminished response by the population in the second challenge of LPS for all proteins ( Figure 7) except NOS2 in both cellular models ( Figure 7B and as seen in the empirical data shown in Figure 4A).

Upon comparing the *in-silico* three-cell-state composition for each of the inflammatory protein, differences, and similarities between BMDM and RAW264.7 cells were visible at 12 and 16 hours of primary *in-silico* stimulus between TNF, IL-6, pro-IL1β and NOS2 ( Figure 8, Figure 9, 3-state). The stimulus length was interpreted based on the empirical results in Figure 1, Figure 2 and Figure 4. For TNF (3-state, Figure 8A), BMDMs had a higher frequency of cells in the NRS than RAW264.7 cells (56% versus 48%) but despite this both maintained a proportion of cells in the negative state (24% versus 15%). This was compatible with the possibility of a fraction of cells remaining negative but capable of responding at later timepoints. In the case of TNF, when the negative state to NRS ratio is calculated in BMDMs, about 40% of phenotypically negative cells can respond to LPS while in RAW264.7 cells this decreases to 30% suggesting that RAW264.7 cells may show greater sensitivity to becoming TNF+ later into the stimulus. In an opposite manner, pro-IL-1β RAW264.7 cells have negative to NRS ratio less than 10% at 16 hours (three-state, Figure 8B) while the same ratio is 25% in BMDMs. While this could be due to the large difference in positive pro-IL-1β cells in RAW264.7 versus BMDM, the model suggested that up to 10% BMDMs remained antigen (LPS)-responsive. Interestingly, the three-state NoRM model suggested similar IL-6 dynamics ( Figure 9A). 3-state model predictions for NOS2 suggested that both BMDMs and RAW cells maintain only about 2% of cells in a negative state post primary or secondary stimulus post-secondary stimulus ( Figure 9B). The above observations demonstrated differences between the two cellular models and their responsiveness to LPS.

While the three-state model was sufficient to explain our experimental data points, it did not differentiate between temporary non-responsiveness and permanent epigenetic cessation of activity ( Seeley & Ghosh, 2017). To explore how these states might vary between proteins and cell types, we also analysed the four-state representation of the NoRM model ( Figure 8, Figure 9, three-state). The NRS to NRPS ratio varied greatly between different proteins after the primary (12hr for BMDM and 16hr for RAW264.7) and secondary (24hr primary+12hr/16hr secondary for BMDM and RAW264.7 respectively) dose of LPS ( *in silico*). TNF NRPS frequencies were almost three times higher than pro-IL-1β in both cellular models. On the other hand, IL-6 NRPS frequency was comparable to TNF NRPS frequency in RAW264.7 ( Figure 8). Further, in RAW264.7 cells, NOS2 NRPS frequency was less than 1% even after secondary stimulus while in BMDMs this was 5% ( Figure 9). The increase in NRPS for any single protein over a subsequent stimulation, however, was consistent for all proteins. This suggested that while some proteins switched off faster in single cells over a course of stimulation, if stimulation remained (i.e., until LPS>0 in the model) the system would progress to all cells becoming non-responsive permanently (NRPS) given γ _2_≠0. Furthermore, over the course primary/secondary stimulus (within our modelling timeframe), BMDMs were consistently comprised of similar frequencies in the NRPS community with respect to RAW264.7 cells for TNF, pro-IL-1β and IL-6, with the exception of NOS2 where BMDM communities had higher NRPS frequency.

Taken together, analysis of the NoRM model demonstrated that the existence of one non-responsive macrophage cell state is necessary to explain the observed empirical data. However, a four-state model including distinct reversible and permanently non-responsive macrophage cell states was also compatible with the empirical data and captured differences between a model macrophage cell line (RAW264.7 cells) and primary macrophages (BMDMs).

## Discussion

Heterogeneity is a hallmark of immune cell populations ( Guilliams *et al.*, 2018; Papalexi & Satija, 2018; Sallusto & Lanzavecchia, 2009; Satija & Shalek, 2014). Understanding the mechanisms driving this heterogeneity can reveal how it can be modulated to prevent immunopathology or boost immunity when necessary ( Davenport *et al.*, 2016; Gogos *et al.*, 2000; Hotchkiss *et al.*, 2013; Rittirsch *et al.*, 2008). In this context, we developed a mathematical model to explore heterogeneity in TNF, IL-6, pro-IL-1β, and NOS2 expression during primary and secondary macrophage responses to LPS. LPS-induced hypo-responsiveness is a physiologically relevant effect in in sepsis and is associated with increased mortality ( Biswas & Lopez-Collazo, 2009). Measuring protein levels of selected key inflammatory mediators using BFA is a limitation of our study. Further studies using the NoRM mathematical model framework and single cell proteomics and transcriptomics can be used to define the key molecular features of non-responsive macrophage subsets within a population responding to antigen *in vitro* and *in vivo* and the molecular regulators driving transitions between responding and non-responding macrophage communities.

We show that single cells show considerable heterogeneity in production and co-expression of TNF, IL-1β, IL-6, or NOS2, underpinned by functionally distinct non-responsive states. It is of note that although both AIH and non-responsiveness are concepts that have been long used in T cell responses ( Schwartz, 2003; Zhu & Paul, 2010), their application and understanding in macrophage responses is profoundly lacking. Our results suggest that, at least with regards to TNF, IL-6, pro-IL-1β, and NOS2 protein expression, heterogeneity in terms of community composition is maintained in hypo-responsive macrophage communities despite the overall lower response. Of note, our model indicates that, at least for a subpopulation of cells, the apparent lack of response is reversible. We note that while generating accurate predictions of temporal evolution of protein positivity was not a primary purpose of the NoRM model, it provides a framework to which linear or non-linear constraints to *μ* (LPS co-efficient) and δ (LPS decay) can be added to model generalised protein positivity at phenomenological levels. This would allow to model primary and secondary effects at objective level generating simple parameters to test in laboratory experiments.

Identifying molecular mechanisms that favor or repress the generation of permanently non-responsive macrophage population can have far-reaching implications for treatment and understanding of infectious, inflammatory, and autoimmune diseases.

Both our empirical and theoretical analysis of macrophage AIH highlighted differences between RAW264.7 cells and primary BMDMs, in agreement with proteomics and transcriptomics studies comparing BMDMs with macrophage-like cell lines ( Guo *et al.*, 2015; Levenson *et al.*, 2018). Differences in pre-existing genetic heterogeneity and signaling and transcriptional networks between the two cell types are likely sources for these differences. However, there also are notable similarities between the two cellular models. For example, macrophages that are challenged with LPS for a second time respond through distinctly different community composition trajectories than those observed in cells that respond to LPS for the first time. Similarly, in the four-state NoRM model, for both cell types LPS-induced hypo-responsiveness is associated with an increase in NRPS. This concurs with reports highlighting that non-reversible mechanisms leading to permanent changes within the cell, such as chromatin remodeling, are critical for induction of endotoxin tolerance ( Seeley & Ghosh, 2017). In a biological context, the NRS can be considered as arising from sufficient but temporary effects such as post-transcriptional attenuation of the TLR4 pathway and/or miRNA induced, while the NRPS might represent longer heritable epigenetic modifications ( Chan *et al.*, 2005; Nomura *et al.*, 2000; Quinn *et al.*, 2012; Seeley & Ghosh, 2017; Vergadi *et al.*, 2018). Further studies on molecular mechanisms that favor or repress the generation of permanently non-responsive macrophage population can have far-reaching implications for treatment and understanding of infectious, inflammatory, and autoimmune diseases.

Innate immune responses are underpinned by heterogeneity ( Satija & Shalek, 2014; Shalek *et al.*, 2013). This is most notable in the high transcriptional variability of cytokines, such as TNF, IL-1β, and IL-6, and their receptors upon stimulus in LPS-stimulated phagocytes ( Hagai *et al.*, 2018). *In vivo*, the source of macrophage population heterogeneity could be driven by developmental, tissue or niche, and activation-associated factors. Furthermore, it can be amplified or suppressed through interaction with other immune or non-immune cells ( Yao *et al.*, 2018). As the main aim of our work was to develop a mathematical model capturing this heterogeneity, our empirical study explored macrophage AIH exclusively *in vitro* using relatively homogeneous starting cell populations. Despite this, we propose that our mathematical model can be used to capture population heterogeneity occurring in more complex macrophage populations or *in vivo*. We speculate that the key concepts revealed by our findings, including AIH-dose dependence, existence of reversible and permanently non-responsive states, and a critical role for transitions between these states as determinants of macrophage function will be relevant to a broad range of pathophysiological contexts in the immune system.

## Data availability

### Underlying data

Figshare: Figure 1: Macrophage community AIH is dependent on LPS dose, https://doi.org/10.6084/m9.figshare.17304641.v3 ( Dey, 2021a)

This project contains the following underlying data:

- FlowJo workspace (WSP) files showing gating strategy along with individual FCS files.

Figshare: Figure 2: Macrophage community AIH kinetics for BMDMs, https://doi.org/10.6084/m9.figshare.17305202.v2 ( Dey, 2021b)

This project contains the following underlying data:

- FlowJo workspace (WSP) files showing gating strategy along with individual FCS files.

Figshare: Figure 3: Altered cytokine production kinetics in RAW264.7 macrophages responding to a second LPS challenge, https://doi.org/10.6084/m9.figshare.17305448.v1 ( Dey, 2021c)

This project contains the following underlying data:

- Raw CSV files containing individual experimental repeat data.

Figshare: Figure 4: Altered AIH kinetics in macrophages responding to a second LPS challenge correlate with hypo-responsiveness, https://doi.org/10.6084/m9.figshare.17305847.v1 ( Dey, 2021d)

This project contains the following underlying data:

- Raw CSV files containing individual experimental repeat data for RAW264.7 and BMDM pies. Also contains FlowJo workspace (WSP) files showing gating strategy along with individual FCS files.

Figshare: Figure 5: BMDMs show a clear hyporesponsive phenotype by flow. Figshare, https://doi.org/10.6084/m9.figshare.18319859.v1 ( Dey, 2022a)

This project contains the following underlying data:

- Raw CSV files containing individual experimental repeat data.

Data are available under the terms of the Creative Commons Attribution 4.0 International license (CC-BY 4.0).

### Extended data

Analysis code available from: https://github.com/jipsi/NoRM

Archived analysis code as at time of publication: https://doi.org/10.5281/zenodo.5851294

License: MIT

### Reporting guidelines

Figshare: Arrive Essential 10 checklist for “Mathematical modelling of activation-induced heterogeneity in TNF, IL6, NOS2, and IL1β expression reveals cell state transitions underpinning macrophage responses to LPS”, https://doi.org/10.6084/m9.figshare.18403988 ( Dey, 2022b)

Data are available under the terms of the Creative Commons Zero "No rights reserved" data waiver (CC0 1.0 Public domain dedication).
