# Supplementary material for: Mathematical modelling of activation-induced heterogeneity in TNF, IL6, NOS2, and IL1β expression reveals cell state transitions underpinning macrophage responses to LPS
Source: Wellcome Open Res. 2022 Jul 25;7:29. Originally published 2022 Jan 28. [Version 2] doi: 10.12688/wellcomeopenres.17557.2 (PMC9411976; doi:10.12688/wellcomeopenres.17557.2)
Supplement: Peer review history

# Peer review: Reviewer response for version 2

**DOI:** 10.21956/wellcomeopenres.19773.r50000

**Published:** 2022-08-25

**Authors:** Yao Lu

**Affiliations:** 1 Dalian Institute of Chemical Physics, Chinese Academy of Sciences, Dalian, China

The manuscript described the experiments to obtain the 4-plexed secretion data from population cells and single-cells with primary and secondary LPS treatment, using two in vitro cellular models (bonemarrow-derived macrophages or BMDMs, and RAW 264.7 cells). They showed that macrophage community AIH (activation-induced heterogeneity) depends on LPS dose and that altered AIH kinetics in macrophages responding to a second LPS challenge underpin hypo-responsiveness to LPS. The observation can be explained by a mathematical three-state model or four-state model. The topic is exciting and important, which could lead to a deep understanding of innate immune responses. I only have several suggestions:

1. I wonder how the single-cell polyfunctionality is distributed within macrophage cells under different treatment conditions, which can be reanalyzed using the data shown in Figures 1B, 2&4.
2. How to identify and verify the existence of NRPS using current experiment data is not clear.
3. The comparisons were made between different cell samples in this study, which lost the dynamic information from the same single-cells at different times and under different treatments. In the future, it would be very interesting to track the single-cell secretion information longitudinally to reveal the changes and connections between different immune cell phenotypes.
4. There should be a space between numbers and units.

Is the work clearly and accurately presented and does it cite the current literature?

Yes

If applicable, is the statistical analysis and its interpretation appropriate?

Yes

Are all the source data underlying the results available to ensure full reproducibility?

Yes

Is the study design appropriate and is the work technically sound?

Yes

Are the conclusions drawn adequately supported by the results?

Partly

Are sufficient details of methods and analysis provided to allow replication by others?

Yes

Reviewer Expertise:

single-cell analysis technologies, microfluidics,

I confirm that I have read this submission and believe that I have an appropriate level of expertise to confirm that it is of an acceptable scientific standard.

# Peer review: Reviewer response for version 2

**DOI:** 10.21956/wellcomeopenres.19773.r51673

**Published:** 2022-08-09

**Authors:** Mark Coles, Miles Weatherseed

**Affiliations:** 1 Kennedy Institute of Rheumatology, University of Oxford, Oxford, UK; 2 University of Oxford, Oxford, UK

I have no further comments, I thank the authors for doing an excellent job and responding to my comments.

Is the work clearly and accurately presented and does it cite the current literature?

Yes

If applicable, is the statistical analysis and its interpretation appropriate?

Yes

Are all the source data underlying the results available to ensure full reproducibility?

Partly

Is the study design appropriate and is the work technically sound?

Yes

Are the conclusions drawn adequately supported by the results?

Yes

Are sufficient details of methods and analysis provided to allow replication by others?

Partly

Reviewer Expertise:

Cellular, Systems and Mathematical Immunology

We confirm that we have read this submission and believe that we have an appropriate level of expertise to confirm that it is of an acceptable scientific standard.

# Peer review: Reviewer response for version 1

**DOI:** 10.21956/wellcomeopenres.19414.r49267

**Published:** 2022-04-05

**Authors:** Mark Coles, Miles Weatherseed

**Affiliations:** 1 Kennedy Institute of Rheumatology, University of Oxford, Oxford, UK; 2 University of Oxford, Oxford, UK

**Summary:** The authors generate 3 candidate biological systems models and use LPS challenge data to determine the most appropriate. They conclude that either their 3-state or 4-state model can explain the observed hypo-responsiveness effect, but note that the 3-state model is potentially more appropriate since it is more parsimonious. 

**Specifically, the authors might consider:**

1. The system of ODEs (Equation S8 – S10) is incorrect and needs amending.
2. There is a reference to a Supplementary Information section (e.g. supplementary Figure 1C) but I cannot see this attached to the paper or on figure share.
3. The detail provided on the mathematical modelling was insufficient for the reader to reproduce this research for themselves, specifically:
  - a. Parameter choices and distributions were not well-justified. This is important for model confidence.
  - b. The choice of parameter delta (=0.5) is claimed to not affect the results. This may be the case but is not immediately intuitive. Evidence for this should be provided, probably in the supplementary information. eFAST sensitivity indices might be a sensible choice. The authors might consider using SPARTAN.
  - c. The NoRM model was run using 10^5 – 10^6 sets of parameters. It should be made clear why this is a sufficient number of samples to estimate the parameter distributions.
  - d. Some/all of the best-fit parameter values for the calculation of the AIC values in the table from Figure 6E should be provided. I would be particularly interested to see the magnitude of the gamma2 parameter as the 3- and 4-state models are identical when this is 0.
4. The model seems to peak many hours before the experimental data peaks in Figure 7. This is particularly pronounced for NOS2. Could this observation guide the development of a further model? For instance, one in which there is a delay between the level of LPS and the upregulation of positive cells.
5. The MATLAB code in the repository does not run. I believe that this is because the raw data required for the fitting are not supplied. It is important that the code runs to permit full exploration.

Is the work clearly and accurately presented and does it cite the current literature?

Yes

If applicable, is the statistical analysis and its interpretation appropriate?

Yes

Are all the source data underlying the results available to ensure full reproducibility?

Partly

Is the study design appropriate and is the work technically sound?

Yes

Are the conclusions drawn adequately supported by the results?

Yes

Are sufficient details of methods and analysis provided to allow replication by others?

Partly

Reviewer Expertise:

Cellular, Systems and Mathematical Immunology

We confirm that we have read this submission and believe that we have an appropriate level of expertise to confirm that it is of an acceptable scientific standard, however we have significant reservations, as outlined above.

# Response

**Published:** 2022-07-05

**Authors:** Shoumit Dey

We thank our reviewers for their comments which have really helped us to streamline our model and, as a result, interpret model outputs better. Please see our replies (in italics) to the reviewers questions (in bold).

**1. The system of ODEs (Equation S8 – S10) is incorrect and needs amending.**

*We apologise. We agree that the equations S8 and S9 were incorrect with a wrong minus sign and missing a term. The ODEs used in the numerics are correct (lps_dynamics_3state2.m) called within execute_ode_ssa_check.m to generate **Figure 6D**; Equations S8 and S9 have now been updated in the revised manuscript. Please note that some mistakes arose in the translation of our submitted manuscript into the reviewed form; it appears that some erroneous mathematical notation was introduced after the document left our control.*

**2. There is a reference to a Supplementary Information section (e.g. supplementary Figure 1C) but I cannot see this attached to the paper or on figure share.**

*Thank you for noticing this, ‘supplementary Figure 1C’ should be ‘Figure 1C’. In addition, there is another mention of the ‘supplementary section’ within the Modelling Process. These have both been corrected.*

**3. The detail provided on the mathematical modelling was insufficient for the reader to reproduce this research for themselves, specifically:**

**a. Parameter choices and distributions were not well-justified. This is important for model confidence.**

*Parameter sets were randomly generated, where each of the parameter (α, β, β _2_, ��, �� _2_) was assumed to be sampled independently from a uniform U[0.01,1] distribution. Further, parameter sets were also sampled from a normal and gamma distribution N(0.03, 0.01) and G(0.68,0.2). Please note we have replaced sampling from the negative binomial distribution to gamma to include overdispersed but non-integer values. The parameterisation of the gamma distribution was chosen to remain faithful to the distribution shape of the negative binomial sampling previously used in version 1 of the manuscript. The same changes have been made in the code file **fit_2_challenge_time_points_param_pies_test.m***

*In the latest version of the manuscript, the above has been re-written under **Methods**, sub-section **Mathematical Modelling** **.***

**b. The choice of parameter delta (=0.5) is claimed to not affect the results. This may be the case but is not immediately intuitive. Evidence for this should be provided, probably in the supplementary information. eFAST sensitivity indices might be a sensible choice. The authors might consider using SPARTAN.**

*The parameter delta represents a decay rate, and our initial choice of delta = 0.5 d ^-1^ represents a half-life of LPS of around one day, which is consistent with the experimental protocols and the observed effects of LPS manipulations. Further refinement of the choices of mu and delta would require finer temporal resolution in the empirical work. In addition, the parameter mu only appears in the combination (mu L) and scales the effect of LPS on the transition from N to P; the models were initially run with mu chosen (arbitrarily) to be 10 for the randomly generated parameter sets for (Q1) detailed above. The results were checked qualitatively by then allowing mu = 0.1, 1, 10 and 100; in all cases the qualitative results were unchanged, but we acknowledge that the exact protein-specific value of mu is a factor that may merit further investigation at higher temporal resolution. As such, in the revised version we have now used only a fixed value of mu (=10) to run the model for both the 3-state and 4-state models. This has resulted in small changes in the output of the above models and we have edited this in the text and included updated Figures 7, 8 and 9.*

*As per the reviewer’s suggestion, we then used SPARTAN’s eFAST approach to generate summary statistics for each parameter at a time to generate first order and higher order sensitivity indices for our model outputs, positive, negative, non-responsive and non-responsive permanent species. All 4 states were considered for sensitivity analysis for the inclusion of all model parameters. In summary, our analysis showed that the appearance of each of the four species was indeed dependent on the rate parameters and less so on mu or delta. The additional analysis is now included as Figure 6 panel E. The parameters mu and delta taken together can indeed be used to fine-tune the kinetics of each of the proteins modelled here. While this was not the primary objective of this NoRM model but as the reviewer has correctly pointed out in Q4 that these parameters can be used to develop the model further.*

**c. The NoRM model was run using 10^5 – 10^6 sets of parameters. It should be made clear why this is a sufficient number of samples to estimate the parameter distributions.**

*We have added Figure 6 panel F to show the rationale for selecting a sufficient number of parameter sets to show how choosing the number of parameter sets affects the average rmsd of the fit to empirical data.*

**d. Some/all of the best-fit parameter values for the calculation of the AIC values in the table from Figure 6E should be provided. I would be particularly interested to see the magnitude of the gamma2 parameter as the 3- and 4-state models are identical when this is 0.**

*We have added in Figure 6 panel H, best-fit parameter values for TNF both for the 3 and 4 state models as an example. All the best fit parameter values are now included with the code in the Github repository (https://github.com/jipsi/NoRM/tree/master/results)*

**4. The model seems to peak many hours before the experimental data peaks in Figure 7. This is particularly pronounced for NOS2. Could this observation guide the development of a further model? For instance, one in which there is a delay between the level of LPS and the upregulation of positive cells.**

*Please see our answer to Q3b and in particular the need for finer temporal resolution in future empirical work. Indeed, a protein-specific mu with the inclusion of delay may be used to develop a model that can explain protein-specific kinetics. However, in this work, our main aim was to check if non-responsive states could indeed model the hyporesponsiveness of a cell’s ability to make protein, and if such a phenomenon is best explained by a transitional non-responsive state and/or by a permanently non-responsive state.*

**5. The MATLAB code in the repository does not run. I believe that this is because the raw data required for the fitting are not supplied. It is important that the code runs to permit full exploration.**

*We apologise. The raw data for the fitting was provided in the repository but the best fit parameter sets were not. We have now added these. It is possible that the code did not run due to this reason. Please start at execute.m and set the value of the parameter ‘search’ to 0. The code should navigate to NoRM/results/’cytokine’/ (please see here - https://github.com/jipsi/NoRM/tree/master/results) and pick up the best fit parameters to print the graphs.*

*In an event when the above does not work, please re-run execute.m after resetting the parameter ‘search’ as 1. This will force the code to search for best fitting parameters from a list of randomly generated parameter sets as described in the **Methods.***
